# Forecasting tool of hospital demand during heat periods: a time-series regression study in Bern, Switzerland

**DOI:** 10.1136/bmjph-2025-004559

**Published:** 2026-08-11

**Authors:** Laura Di Domenico, Martin S Wohlfender, Wolf E Hautz, Ana Maria Vicedo-Cabrera, Christian Althaus

**Affiliations:** 1 Institute of Social and Preventive Medicine, University of Bern, Bern, Switzerland; 2 Data Science Institute, Hasselt University, Hasselt, Belgium; 3 Multidisciplinary Center for Infectious Diseases, University of Bern, Bern, Switzerland; 4 Graduate School for Cellular and Biomedical Sciences, University of Bern, Bern, Switzerland; 5 Department of Emergency Medicine, Inselspital University Hospital, Bern, Switzerland; 6 Oeschger Center for Climate Change Research, University of Bern, Bern, Switzerland

**Keywords:** Epidemiology, Epidemiologic Factors, Emergencies

## Abstract

**Introduction:**

Heat significantly impacts human health by causing heat strain or exacerbating pre-existing conditions. Hospitals may suffer a higher healthcare demand during intense heat periods, especially if climate change continues to increase the severity and frequency of heatwaves. Anticipating episodes of higher hospital demand would allow better resource planning and quality of care.

**Methods:**

We developed a real-time forecasting tool of daily hospital demand (specifically, all-cause emergency room visits (ERVs)), which accounts for the impact of heat. Our tool is based on a regression model integrating temperature-ERV function with autoregressive terms and other temporal trends. The model can (1) quantify the association between the number of hospital visits and temperature based on historical data and (2) provide accurate short-term forecasts of the daily ERV based on temperature values expected for the upcoming days. As a case study, we used data from Bern University Hospital for the summers of 2014–2022, and mean temperature per day as an indicator of heat exposure.

**Results:**

Temperature–ERV relationship exhibited a non-linear shape. We found that, with respect to the mean temperature of minimum risk of 15 °C, there were approximately 6 (95% CI 2 to 10) additional ERVs when mean temperature was around 25°C, corresponding to a 3% increase in summer 2022. The estimated variation increased for mean temperature above 25 °C but with large uncertainty. We also found that our model showed higher accuracy at forecasting hospital demand during periods with particularly hot days, compared with a model neglecting temperature. Our forecasting tool is implemented in a user-friendly R Shiny app, allowing for application to new datasets.

**Conclusions:**

We found a robust association between ambient temperature and visits to the emergency department in a Swiss hospital. Our findings suggest that including temperature can increase the accuracy of predictions for hospital demand during summer.

**WHAT IS ALREADY KNOWN ON THIS TOPIC**

- Heat significantly impacts human health, leading to increased morbidity and mortality risks.
- Intense heatwaves can lead to high healthcare demand, potentially overcrowding emergency departments and decreasing quality of care.

**WHAT THIS STUDY ADDS**

- We developed a real-time forecasting tool for daily all-cause emergency room visits (ERVs) during summer, accounting for the impact of heat.
- In a case study in a major hospital in Switzerland, we estimated a higher number of ERVs during periods with higher temperature.

**HOW THIS STUDY MIGHT AFFECT RESEARCH, PRACTICE OR POLICY**

- We implemented our tool in a user-friendly web application, allowing for replication of the analysis to new datasets.
- Hospitals can use this forecasting tool to anticipate increased healthcare demand during heatwaves.

## Introduction

 Exposure to high temperatures significantly impacts human health, causing heat-related physiological stress or exacerbating pre-existing conditions such as cardiovascular diseases and respiratory disorders like asthma.^1^ Extreme heat episodes have been increasingly recognised as a serious public health threat, especially in the context of climate change with frequency and severity of heatwaves expected to increase in the future.^2^ Furthermore, the increasing age of many populations aggravates the problem as older people are substantially less heat tolerant than younger ones.^3^ However, the potential impact of climate change on healthcare workers and the quality of care remains unclear. Numerous studies quantified the association between high temperature and increased morbidity, including hospitalisations and visits to the emergency room.^4–7^ Therefore, healthcare infrastructure may suffer under future scenarios of high warming with periods of very intense heat. This has become even more relevant, since recent public health crises such as the COVID-19 pandemic showed how vulnerable the healthcare system can be in periods of extremely high demand. Thus, forecasts of hospital demand during heat periods can be useful for resource planning, especially for emergency departments (EDs) to avoid the ubiquitous problem of overcrowding and reduced quality of care.^8,9^

By using standard methods in health impact assessment, recent epidemiological investigations estimated the mortality and morbidity burden due to heat in different study settings.^10,11^ However, very few studies exploited this relationship to produce short-term forecasts.^12^ In this study, we developed a forecasting tool of the number of hospital visits during summer, accounting for the impact of heat. Our tool is based on a regression model that combines time-series predictors (eg, autoregressive terms, weekly pattern) and daily temperature through a distributed lag non-linear model. As a case study, we applied our forecasting model to predict the number of emergency room visits (ERVs) during summer in Bern University Hospital, one of five first-level university general hospitals of Switzerland. We obtained the exposure–response function empirically using observed weather and health data and assessed the accuracy of short-term forecasts of hospital demand, both including and omitting temperature in the model. We implemented our tool in a user-friendly R Shiny app that can be easily used and extended in other case studies.

## Materials and methods

### Data sources

#### Hospital data

We used routinely collected individual-level electronic health records from the Bern University Hospital in the city of Bern, Switzerland. We extracted the daily number of patients visiting the ED for any cause (all-cause ERVs). The number includes both ambulatory patients (discharged within the day) and patients admitted to the hospital for at least one night. We considered historical data for the period ranging from 2014 to 2022. We restricted our analysis to the warmest months of the year, defined from 1 June to 31 August. The ED in Bern is a tertiary, university affiliated ED that sees around 55 000 patients per year, making it one of the largest in the country. Around 35% of patients are hospitalised. On average, 45% of patients presenting to the ED are female, patients’ mean age is 49.5 (SD 21.2) years. Patients younger than 16 years are seen in a separate paediatric ED. Around 40% of all patients self-admit, while another 20% are brought in by ambulance or helicopter. The remainder of the patients result from transfers to the ED from external hospitals or general practitioners. Emergency medicine in Switzerland is easily accessible to the population, with costs covered by mandatory healthcare insurance, subject to the insured individual’s chosen deductible.

#### Temperature data

We used historical data of daily mean temperature, registered at the Zollikofen weather station between 2014 and 2022, the closest station to the city of Bern. Data were requested through the IDAweb portal,^13^ maintained by Meteo Swiss. To evaluate forecasts, we first used the observed temperature on the test set. To simulate a real-time application, we then used forecasted temperature data provided by Meteo Swiss. To ensure comparison of the temperature grid data with the observed historical data, we considered a grid point in the surroundings of the Zollikofen measurement station and bias-corrected for altitude difference. Besides mean temperature per day, we also considered other temperature indicators (ie, daily and nocturnal means and minimum and maximum temperature) in a sensitivity analysis (online supplemental figure S7).

### Statistical analysis

#### Statistical model

Our model included both autoregressive terms and explanatory variables as model predictors.^14^ The outcome variable was the daily number of ERVs. In short, the model included the following variables: an intercept, previous ERVs up to 7 days in the past, day of the week and holiday effect, a level shift in the 2020–2022 period to account for COVID-19 effect, and lagged temperature values from lag 0 to lag 3. This allows the model to capture short-term effects of temperature and harvesting effect. For temperature, we used an unconstrained distributed lag non-linear model,^15^ meaning that each lagged temperature was modelled with a non-linear function (natural splines). The full mathematical description of the model is presented in online supplemental information, along with model coefficient estimates (online supplemental figure S2) and residual diagnostics (online supplemental figures S4 and S5). We then computed the cumulative exposure–response function by aggregating over the lags. We did not put constraints in the lag-response dimension, meaning that each lagged temperature was modelled independently. This choice was adopted to ease implementation for forecasting purposes. We fitted the regression model assuming a Gaussian distribution. We found that the shape of the exposure-response function estimated from our dataset was not sensitive to the choice of the family (Gaussian or quasi-Poisson, online supplemental figure S8).

#### Model calibration and evaluation

We estimated the exposure–response function linking ERV counts and temperature by training the model on data from 2014 to 2021 (training set: 1 June 2014–31 August 2021). We used data from summer 2022 as a test set (test set: 1 June 2022–31 August 2022). We also carried out sensitivity analysis with cross-validation (ie, using a different year as test set, and the remaining years as training set) to check the robustness of the shape of the exposure–response curve (online supplemental figure S6). To evaluate model accuracy, we computed the root mean square error (RMSE) between the 1-step ahead model predictions *H(t)* and the observed data *H(t)* for each day t. We computed the RMSE on the training set and on the test set. Model accuracy was then compared with a model without temperature and basic last-observation-carried-forward (LOCF) models with a 7-day lag or with a 1-day lag (ie, the prediction *H(t)* for day t is equal to *H (t–7)* or *H (t–1)*, respectively).

#### Forecasts

We generated 21-step ahead forecasts, focusing on 7-day forecast windows of 1 (1–7-step ahead forecasts), 2 (8–14-step ahead forecasts) and 3 weeks (15–21-step ahead forecasts) ahead for the evaluation. We generated point predictions and prediction intervals (PI) at 50% and 90%. We evaluated forecast errors of the point predictions using RMSE over the forecast window. We compared the average RMSE across all forecast windows obtained using a model with temperature or without temperature. Comparison of the two models was also framed as a function of the average temperature in the forecast window to determine which model performed better specifically over forecast windows with a high average temperature. To test the robustness of the comparison and out-of-sample model performance, we carried out a cross-validation analysis generating forecast not only for summer 2022 but also for any year as a test set (from 2014 to 2022), excluding the year of the test set from the training data. Finally, we generated the forecasts retrospectively on the test set (eg, summer 2022), using available historical data for temperature; to simulate a real-time use of the forecasting tool, we used forecasted temperature data for summer 2022 and compared model outcomes with the results obtained using observed temperature data.

## Results

### Estimation of the association of ERV with temperature

**Figure 1:**
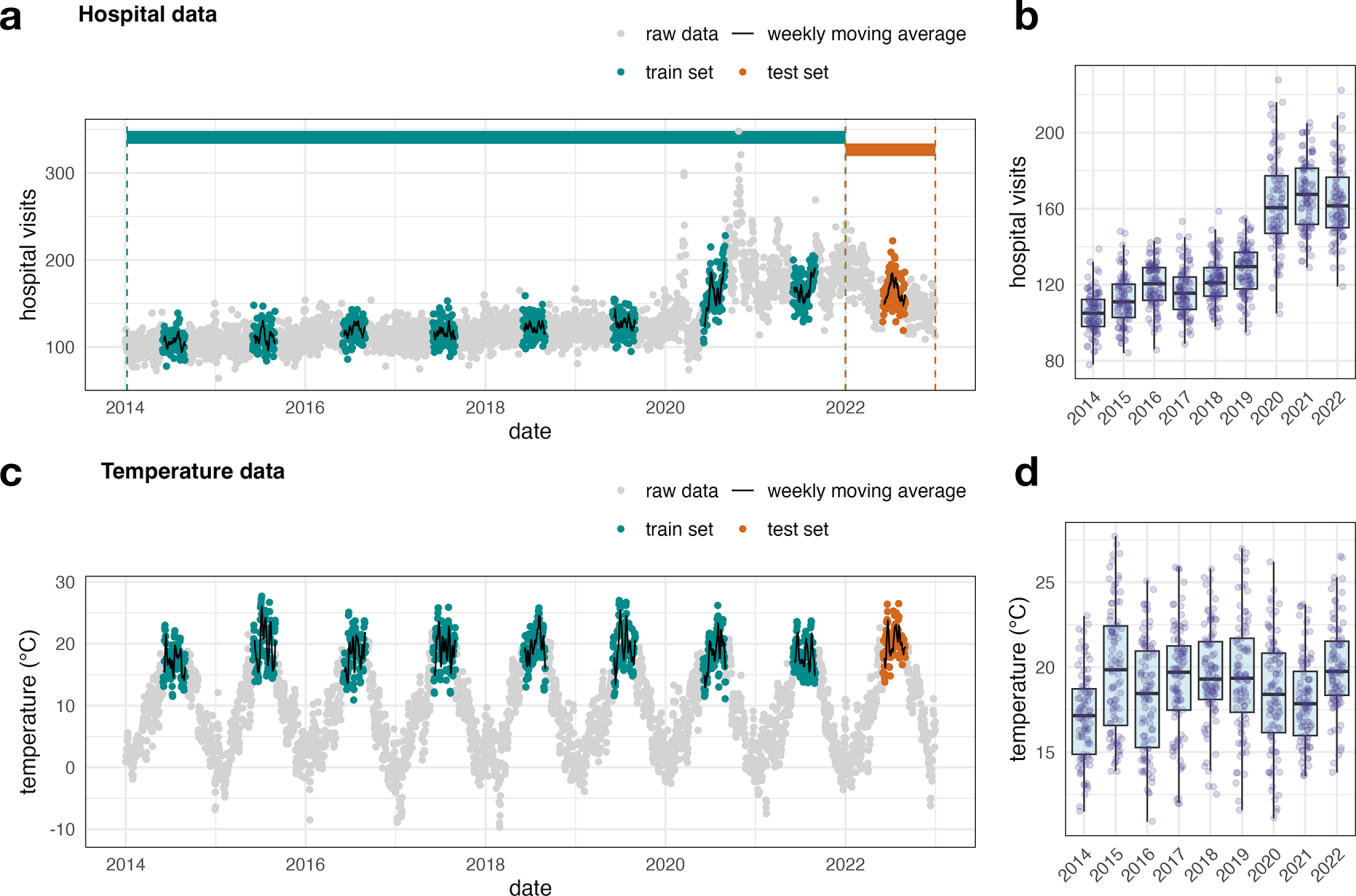
Data. (**a**) Time series of daily emergency room visits (ERVs). (**b**) Boxplot (median and IQR) showing the distribution per year. (**c**) Time-series of the observed mean temperature per day, expressed in °C. (**d**) Boxplot showing the distribution per year. In (**a**) and (**c**), grey dots represent the full time-series, coloured dots represent summer data used for the analysis (from 1 June to 31 August). Green and red datasets indicate the training set and the test set, respectively.

We used historical data on ERV and mean temperature per day from 2014 to 2022 (figure 1). The time series of ERV showed a significant level shift in the period ranging from 2020 to 2022, during the COVID-19 pandemic (figure 1a,b). The periods registering the largest number of days with particularly high temperature (>25 °C) were summer 2015 and 2019 (figure 1c,d, online supplemental figure S1).

We fitted our model to a training set ranging from 2014 to 2021. Model predictions (1-step ahead) for each year of the training set are shown in online supplemental figure S3. The Akaike information criterion (AIC) showed that the model including temperature had some support over the alternative model without temperature, however the evidence was not strong (ΔAIC=3.1, online supplemental table S1). We computed the RMSE between the 1-step ahead model predictions and the observed data on the training set and the test set (summer 2022). We found that the error of our model was much lower than the error of last-observation-carried-forward models. The error was similar to a model with the same set of predictors but removing temperature. These results are summarised in online supplemental tables S2 and S3.

**Figure 2:**
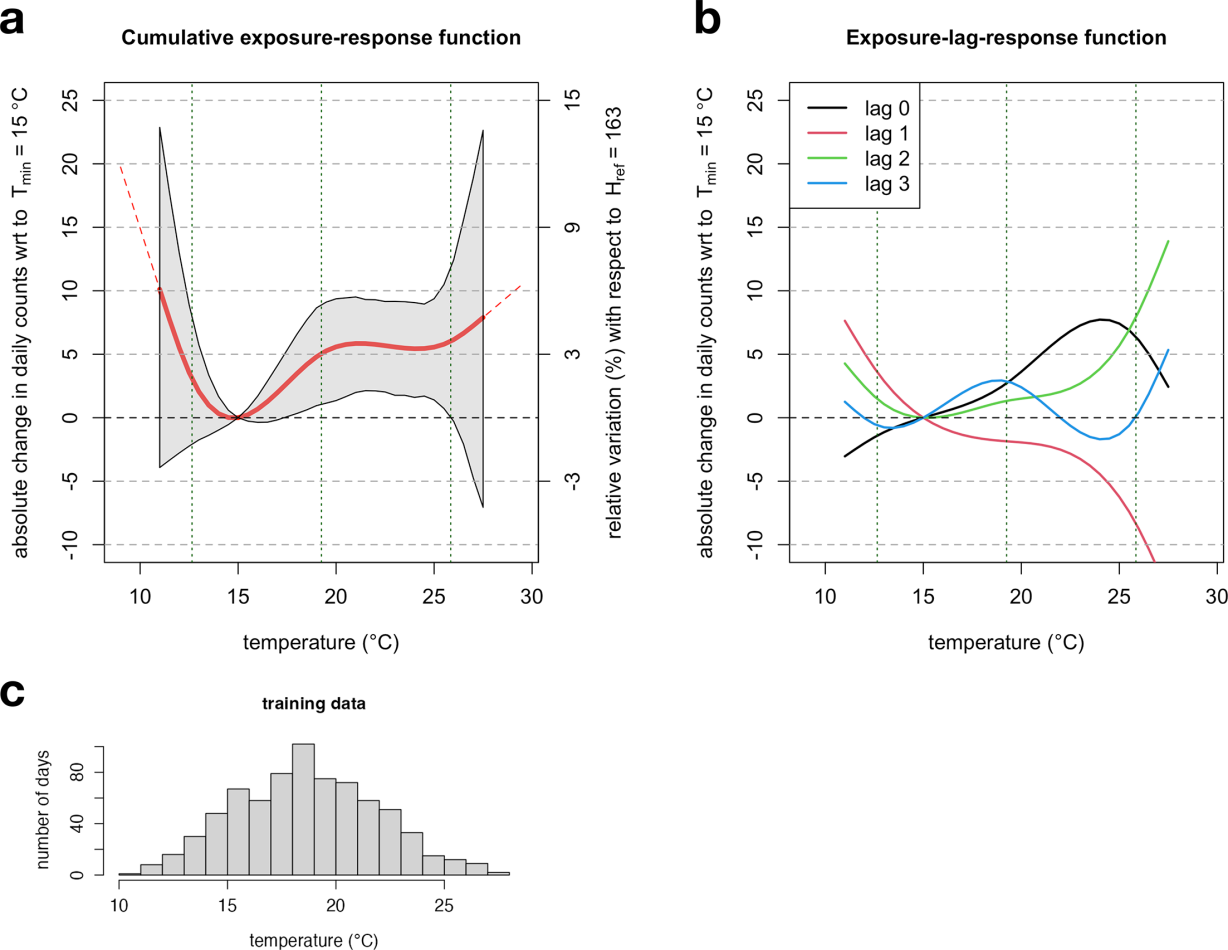
Estimated non-linear association with mean temperature per day. (**a**) Cumulative exposure-response function, showing the absolute change in daily hospital visits as a function of temperature, with respect to a reference temperature of minimum risk of 15 °C. On the right y-axis, relative variation with respect to the average number of visits registered during summer 2022 (test set). (**b**) Lag-exposure-response function for different lags. In (**a**) and (**b**), vertical dashed green lines indicate the position of the internal knots used to define the non-linear spline functions. (**c**) Histogram of the mean daily temperature values observed in the training set.

We computed the cumulative exposure–response function (figure 2a), measuring the non-linear association between ERV and temperature, by aggregating the effects of lags up to 3 days in the past (figure 2b). We found that, compared with a mean temperature of minimum risk of 15 °C (as estimated by the model), there were approximately 6 (95% CI 2 to 10) additional ERV when mean temperature was between 20°C and 25°C. This number corresponded to a 3% (95% CI 1% to 6%) relative increase over the average number of visits registered during summer 2022 (during COVID-19 period) or 5% (95% CI 2% to 9%) over the average observed before COVID-19. The estimated median variation increased for extremely high temperatures (28°C), although with large uncertainty due to the low occurrence of such values in the training set (figure 2c). We found that the shape of the exposure–response function was stable after cross-validation on the training set (online supplemental figure S6).

### Forecasts

**Figure 3:**
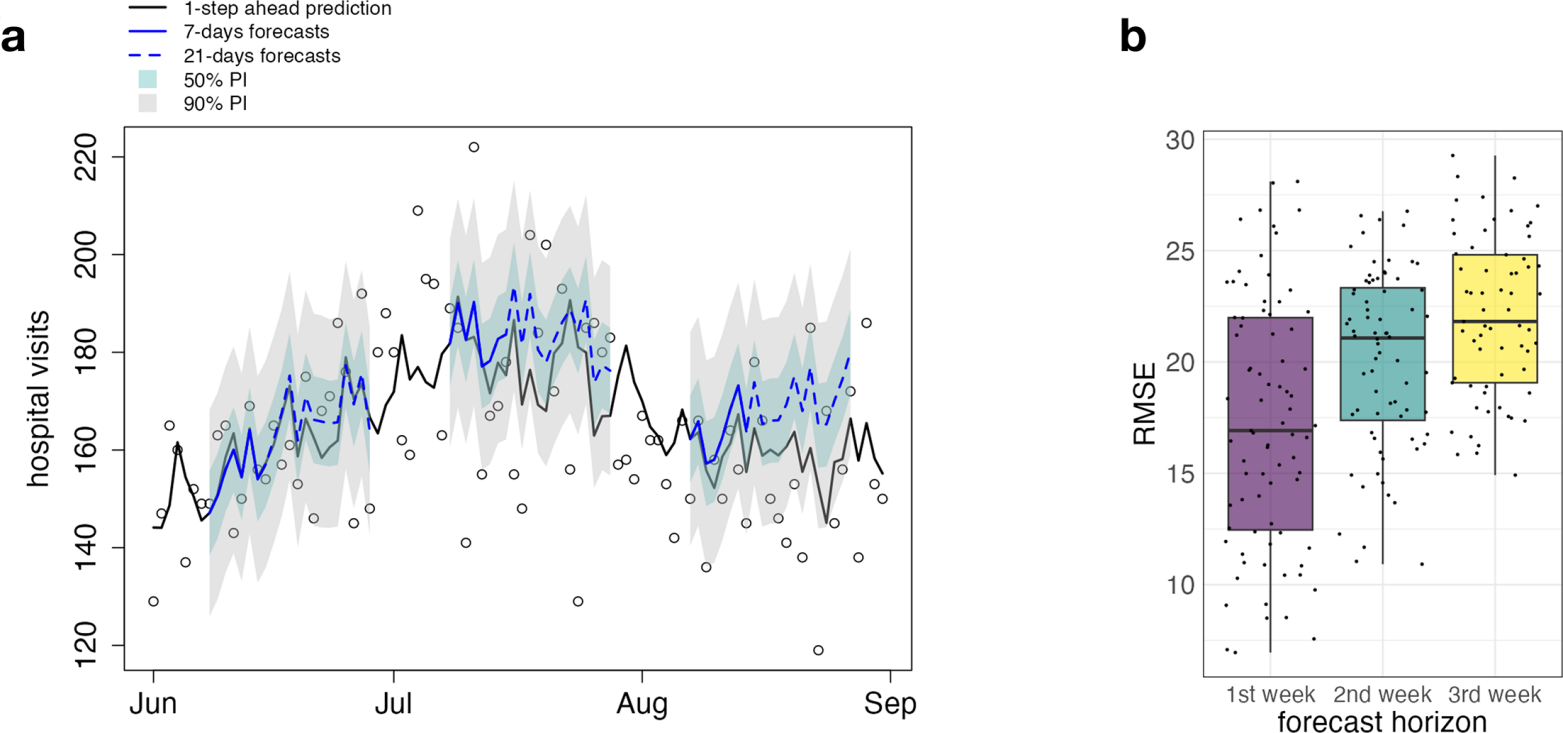
Forecasts of daily emergency room visit (ERV) with different time horizons. Example using summer 2022 as test set. For each given forecasting horizon (1, 2 or 3 weeks), we consider time windows with different starting dates, spanning from 8 June up to the end of August. (**a**) Daily number of hospital visits (specifically, emergency-room visits). Three forecast windows (with starting date in June, July or August) are shown as examples. Blue lines show the estimated model prediction, along with 50% and 90% prediction intervals (PI) shown with shaded areas. In black, we show the 1-step ahead model prediction. (**b**) Forecast error evaluated over a period of 7 days, in the first, second or third week from the starting date. The boxplot indicates the median and the interquartiles.

We used our model to produce forecasts of daily ERVs up to 3 weeks ahead for the testing period (2022), and retrospectively evaluated forecast errors using the observed data as reference (figure 3). The model is able to capture changes in trends of patient visits (figure 3a). The model is more precise in predicting the pattern of hospital visits in the upcoming week, while errors become increasingly larger when evaluating predictions 2 or 3 weeks in the future (figure 3b).

We also generated forecasts of daily ERV using forecasted temperature data, to simulate the use of the tool in real time. We found that the forecast errors remained similar when replacing observed temperature data with forecasted temperature data (online supplemental figure S12), provided that the two sources are in good agreement (online supplemental figure S10). We also found that integrating the uncertainty in the forecasted temperature data (with variations around 2 °C, online supplemental figure S9) in the hospital forecasting model did not significantly increase the width of the PIs (online supplemental figure S11).

**Figure 4:**
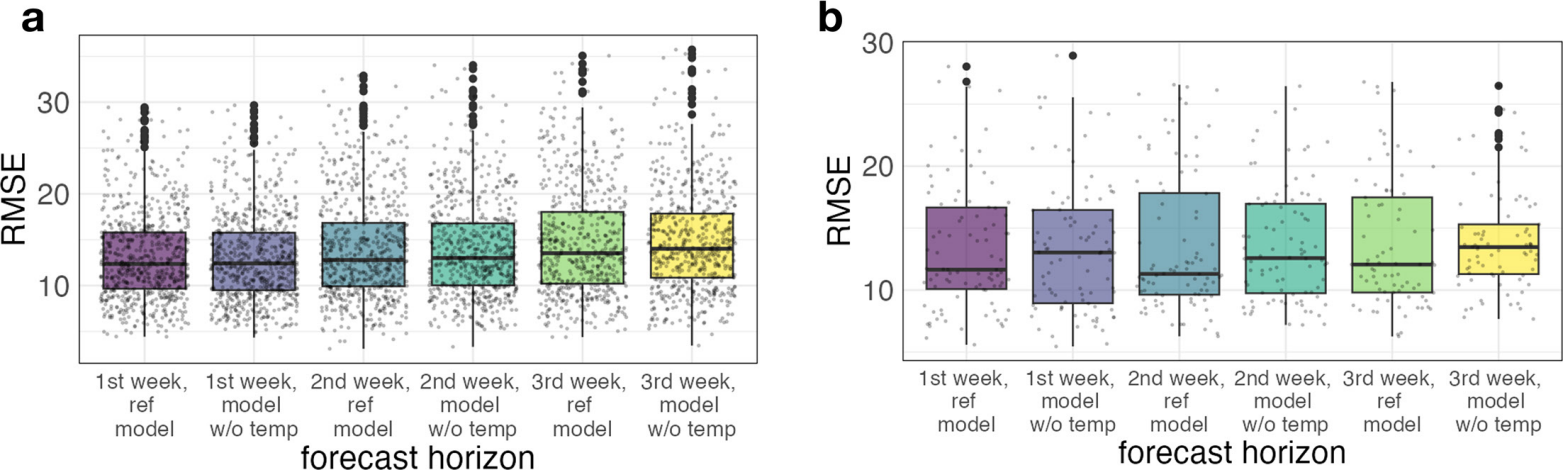
Comparison of forecast errors for a model with or without temperature. We generate forecasts for each year (from 2014 to 2022), excluding the year of the test set from the training data (cross-validation). For each summer, we consider 65 time-windows of 21 days, with a starting date spanning from 8 June to 11 August. We generate 21-step ahead forecasts, and evaluate the root mean square error (RMSE) on three forecast horizons (first week, 1–7 step ahead forecasts; second week, 8–14 step ahead forecasts; third week, 15–21 step ahead forecasts). (**a**) RMSE for all forecast windows. (**b**) RMSE only for forecast windows with average temperature above 22 °C. Results are illustrated with boxplots (box: median and IQR; whiskers based on Q1 − IQR x 1.5 and Q3 + IQR x 1.5). Values are reported in online supplemental table S4.

Finally, we aimed to assess whether the proposed model provided more accurate forecasts compared with a model neglecting temperature. To increase the sample sizes, we generated forecasts ranging from 2014 to 2022, using models trained on all data except the year of the forecasts (ie, using cross-validation on the training set). We compared the RMSE of the forecasts with respect to a model neglecting temperature. First, we considered all forecast windows. We found that the median RMSE was similar for the first week of prediction, and slightly smaller for the second or third week of predictions with respect to a model neglecting temperature (figure 4a). Then, by selecting only the forecast windows for which the average temperature was quite high, we found that the median RMSE obtained with the proposed model was lower than a model without temperature (figure 4b). This is illustrated in figure 4b using a threshold of 22 °C, but this trend was observed for any temperature threshold larger than 20°C. Overall, these results suggest that, although for middle–low temperature values the two models perform similarly, a model including temperature is more accurate when forecasting over periods with particularly hot days compared with a model neglecting temperature.

## Discussion

Here, we present a method and tool to predict future hospital demand in terms of ERVs accounting for the effect of temperature. We estimated the non-linear relationship between temperature and hospital visits, using state-of-the-art methods and historical data, and leveraged this relationship to generate forecasts of expected hospital demand for resource planning. To achieve this, we adopted a novel approach that combines a distributed lag non-linear model with a time-series forecasting framework. In our case study, we found a non-negligible association between observed temperature and ERVs in a major hospital in Bern, Switzerland. Temperature above 20°C corresponded to an increase of at least 3% in the daily ERVs compared with an optimal temperature of reference (estimated at 15°C). From an operational point of view, such an increase could affect hospital bed demand, as patients with heat-related issues are more likely to be hospitalised than average ED patients. The model was able to reproduce historical trends in ERVs. Short-term forecasts (1–3 weeks ahead) showed higher accuracy over forecast horizons with elevated average temperature, compared with a model neglecting temperature, suggesting that our model more effectively captured the impact of heatwaves on hospital demand.

We implemented our model as a user-friendly R Shiny application^16^, facilitating its application to other case studies, and enabling potential real-time use when informed with forecasted temperature data. While we demonstrated our approach using ED data, the model is easily adaptable to other datasets, for example, visits to other hospital wards or patients with specific diagnoses. Additionally, our tool allows the user to estimate the corresponding location-specific relationship between hospital visits and temperature using observed data, which is crucial given that this relationship may vary across settings. Forecasts are then generated using this specific exposure–response function. Finally, our framework allows for testing various temperature indicators. Although mean daily temperature was used in our main analysis, alternative metrics may also be relevant, for example, nocturnal heat which has been found to be associated with increased risks of stroke.^17^

Our study has some limitations. First, the model was trained on a limited number of years, and heatwave episodes were quite rare; more data encompassing extreme heat periods would allow to refine the model estimates. A key assumption of our forecasting tool relates to the exposure–response function used to generate forecasts. This function is estimated on historical data and is assumed to be representative of current and future heat-related risks, meaning that our framework does not account for adaptation to heat or increased heat intolerance in an ageing population. Incorporating an exposure–response function, which evolves over time could help account for potential adaptation mechanisms, but as of today, the required direction and extent of such a model adaptation remains unknown. Another limitation concerns the impact of the COVID-19 pandemic. We assumed that, from 2022 onwards, the average hospital demand returned to prepandemic levels (prior to 2020). This assumption was met for our case study but may not be optimal for all datasets. Forecast errors were assessed using only the point estimates, without considering the full range of uncertainty; future studies could adopt evaluation metrics that explicitly incorporate predictive uncertainty. Finally, while we integrated forecasted temperature data into our model, we did not retrospectively assess the association between ERVs and forecasted temperature values. This was not feasible for our case study, due to the availability of only one summer of forecasted temperature data. Nevertheless, the comparison of forecasted and observed temperature data indicated good agreement, suggesting that bias correction was not necessary.

Our study identifies heat as a relevant driver for ED visits during summer and suggests that inclusion of temperature can increase the accuracy of forecasts of hospital demand during heatwaves. Forecasting tools that account for this effect would be particularly relevant for hospital capacity planning, especially in the context of current projections of climate change, with more frequent and intense heatwaves expected in the future. The forecasting tool for ED visits developed in this study offers a foundation for broader implementation in real-time settings and future integration in the management infrastructure of healthcare facilities.

## Supplementary material

10.1136/bmjph-2025-004559 online supplemental file 1

## Data Availability

Data are available in a public, open access repository.

## References

[1] WHO (2026). Heat and health. https://www.who.int/news-room/fact-sheets/detail/climate-change-heat-and-health.

[2] Lüthi S, Fairless C, Fischer EM (2023). Rapid increase in the risk of heat-related mortality. Nat Commun.

[3] Scovronick N, Sera F, Vu B (2024). Temperature-mortality associations by age and cause: a multi-country multi-city study. *Environ Epidemiol*.

[4] Requia WJ, Vicedo-Cabrera AM, de Schrijver E (2023). Association of high ambient temperature with daily hospitalization for cardiorespiratory diseases in Brazil: A national time-series study between 2008 and 2018. Environ Pollut.

[5] Salvador C, Gullón P, Franco M (2023). Heat-related first cardiovascular event incidence in the city of Madrid (Spain): Vulnerability assessment by demographic, socioeconomic, and health indicators. Environ Res.

[6] Ragettli MS, Vicedo-Cabrera AM, Flückiger B (2019). Impact of the warm summer 2015 on emergency hospital admissions in Switzerland. Environ Health.

[7] Schulte F, Röösli M, Ragettli MSR (2024). Risk, Attributable Fraction and Attributable Number of Cause-Specific Heat-Related Emergency Hospital Admissions in Switzerland. Int J Public Health.

[8] Hoot NR, Aronsky D (2008). Systematic review of emergency department crowding: causes, effects, and solutions. Ann Emerg Med.

[9] Boyle A, Higginson I, Sarsfield K RCEM acute insight series: crowding and its consequences.

[10] Hundessa S, Huang W, Zhao Q (2024). Global and Regional Cardiovascular Mortality Attributable to Nonoptimal Temperatures Over Time. J Am Coll Cardiol.

[11] Alahmad B, Khraishah H, Kamineni M (2024). Extreme Temperatures and Stroke Mortality: Evidence From a Multi-Country Analysis. Stroke.

[12] Mistry MN, Gasparrini A (2024). Real-time forecast of temperature-related excess mortality at small-area level: towards an operational framework. *Environ Res Health*.

[13] MeteoSchweiz Datenportal für lehre und forschung (IDAweb). https://www.meteoschweiz.admin.ch/service-und-publikationen/service/wetter-und-klimaprodukte/datenportal-fuer-lehre-und-forschung.html.

[14] Hyndman & Athanasopoulos (2018). Forecasting: principles and practice (2nd ed).

[15] Gasparrini A, Armstrong B, Kenward MG (2010). Distributed lag non-linear models. Stat Med.

[16] Di Domenico L, Wohlfender MS, Hautz WE (2025). ISPMBern/hospital_forecasting_tool: first release.

[17] He C, Breitner S, Zhang S (2024). Nocturnal heat exposure and stroke risk. Eur Heart J.

