# Supplementary material for: Forecasting tool of hospital demand during heat periods: a time-series regression study in Bern, Switzerland
Source: BMJ Public Health. 2026 Aug 11;4(3):e004559. doi: 10.1136/bmjph-2025-004559 (PMC13475132; doi:10.1136/bmjph-2025-004559)
Supplement: Supplementary file 1 [file bmjph-4-3-s001.docx]

## Supplementary Information

# A forecasting tool of hospital demand during heat periods: a time series regression study in Bern, Switzerland

Laura Di Domenico^1,*^, Martin S. Wohlfender^1,2,5^, Wolf E. Hautz^3^, Ana M. Vicedo-Cabrera^1,4^, Christian L. Althaus^1,2^

*^1^ Institute of Social and Preventive Medicine, University of Bern, Bern, Switzerland*

^2^ *Multidisciplinary Center for Infectious Diseases, University of Bern, Bern, Switzerland*

*^3^ Department of Emergency Medicine, Inselspital University Hospital Bern, Bern, Switzerland*

*^4^ Oeschger Center for Climate Change Research, University of Bern, Bern, Switzerland*

*^5^ Graduate School for Cellular and Biomedical Sciences, University of Bern, Bern, Switzerland*

*corresponding author: laura.didomenico@uhasselt.be

### Table of contents

[Temperature data	2](#_Toc234836306)

[Model description	2](#_Toc234836307)

[Model selection	4](#_Toc234836308)

[Model coefficients, fit and evaluation	5](#_Toc234836309)

[Residual diagnostics	8](#_Toc234836310)

[Exposure-response function with cross-validation	9](#_Toc234836311)

[Other temperature indicators as exposure variable	10](#_Toc234836312)

[Gaussian and quasi-Poisson regression	11](#_Toc234836313)

[Forecast errors	12](#_Toc234836314)

[Observed vs forecasted temperature data	13](#_Toc234836315)

### Temperature data

The seasons registering the largest number of days with mean temperature above 25 °C were summer 2015 and summer 2019, with 9 and 6 days respectively. For context, the 25 °C threshold is currently being used by MeteoSwiss to issue heat warnings in Switzerland.

**Figure S1.** Temperature data. Each plot shows the daily time-series of mean temperature, for each year ranging from 2014 to 2022, from June 1st to August 31st. Days with temperature values exceeding 25 °C are highlighted in red.

### Model description

The model can be summarised as follows

$$H(t) = \beta_{0}+ \sum_{s=1}^{7} \beta_{s}H(t-s) + \sum_{k=0}^{3} f(T(t-k), {\underline{\gamma_{k}}})+\sum_{d} \delta_{d}D_{d}(t) +\eta X_{pandemic}(t) + \epsilon(t)$$

where $H(t)$ is the outcome, i.e. the daily number of ERV on day $t$, and $\epsilon(t)$ represent the error terms. The coefficient $\beta_{0}$denotes the intercept. The autoregressive terms $H(t-s)$ refer to daily observations of hospital visits up to seven days in the past. To model the association with temperature $T(t)$, we used an unconstrained distributed lag non-linear model[^15^](https://www.zotero.org/google-docs/?Jw8ntF). In detail, we used a natural spline function $f$ (i.e., a cubic spline with constraints of linearity beyond the boundary knots), for each $T(t-k)$ from current day $t$ up to $k=3$ days in the past. This allows the model to capture short-term effects of temperature and harvesting effect. For each lag, we used three internal knots for the spline function, i.e the median and 10% and 90% quantiles of the observed temperature range, for a total of four degrees of freedom. Hence, we estimated four parameters ${\underline{\gamma_{k}}}=(\gamma_{k}^{1} ,\gamma_{k}^{2},\gamma_{k}^{3},\gamma_{k}^{4})$ for each lag. We then computed the cumulative exposure-response function by aggregating over the lags. We did not put constraints in the lag-response dimension, meaning that each lagged temperature was modeled independently. This choice was adopted to ease implementation for forecasting purposes. Finally, we included the following control variables for temporal trends. We included seven dummy variables $D_{d}(t)\in\{0,1\}$ to account for the day of the week, distinguishing between regular weekdays ($d=$ Tuesday, …, Friday), Saturday, Sunday and holiday within the week; Monday was used as reference. We included a dummy variable $X_{pandemic}(t)$ which equals to 1 in the period from 2020 to 2022, to capture a level shift during the COVID-19 pandemic. The inclusion of these temporal variables was based on improvement of the Akaike Information criterion (AIC) (Table S1). For sensitivity, we also tested the inclusion of a within-year monthly seasonality, but this did not improve the AIC.

To generate $h$-step ahead forecasts retrospectively on the test set, we adopted the following procedure. Let day $t$ be the start of our forecast window, and let $\hat{H}(t)$ be the 1-step ahead prediction obtained with the model described above. To extend our forecasts, we used $\hat{H}(t)$ as autoregressive term to compute $\hat{H}(t+1)$, using the observed temperature data $T(t+1)$as input, and so on up to $\hat{H}(t+h -1)$. To compute forecasts in real time starting from day $t$, forecasted temperature data $\hat{T}(t+k)$for $k = 0, ... , h-1$ are needed to feed the model.

To propagate uncertainty of Ĥ(t) to the forecast of Ĥ(t+1), we constructed prediction intervals with a Monte Carlo simulation approach, using the following procedure. We generated 500 forecast trajectories. For each trajectory, we first generated the 1-step ahead prediction Ĥ(t). Then, we sampled y(t) ~ N(Ĥ(t), ${\sigma^{2}}_{pred}$) from a normal distribution centered in Ĥ(t) and using as variance ${\sigma^{2}}_{pred}= {\sigma^{2}}_{fit}+{\sigma^{2}}_{res}$, where $\sigma_{fit}$ is the standard error of the fitted value Ĥ(t) and $\sigma_{res}$ is the residual standard error of the model. Finally, we used y(t) as input to forecast Ĥ(t+1). We iterated this procedure for each step in the forecast horizon (i.e. sampling y(t+1) from the predictive distribution of Ĥ(t+1) and use y(t+1) as input for Ĥ(t+2), and so on). After generating 500 forecast trajectories, the final prediction intervals for each time step were estimated by computing the median and quantiles of the forecast trajectories.

### Model selection

We compared our proposed model with variations of the model removing some predictors (temperature, autoregressive terms, weekly pattern, COVID-19 shift) or including additional temporal effects (month seasonality). In this set of models, our proposed model showed the best goodness of fit (lowest AIC). There was strong evidence against a model without weekly pattern, without COVID-19 level shift or without autoregressive terms ($\Delta AIC>$ 20), while the other model including a month indicator scored with $\Delta AIC$ around 2. The model without temperature led to $\Delta AIC$ = 3.1 compared to the proposed model, thus showing some support for the model including temperature, but with weak evidence.

| **Model** | **AIC** |
| --- | --- |
| Proposed model | 5857.7 |
| Proposed model without temperature | 5860.8 |
| Proposed model without day of the week & holiday effect | 5953.6 |
| Proposed model without autoregressive terms | 6124.8 |
| Proposed model without level shift due to COVID-19 | 5880.4 |
| Proposed model with month indicator | 5859.9 |

**Table S1.** Akaike Information Criterion (AIC) for different models.

### Model coefficients, fit and evaluation

Besides the estimated coefficients related to temperature, we can also extract coefficients related to other predictor variables, in order to provide some interpretability of the model (Fig. S2). Our model included seven autoregressive terms, i.e. the daily number of hospital visits observed in the last seven days. We found that the corresponding coefficients decrease the longer the lag. This suggests that daily counts observed in less recent days play a smaller role in predictions with respect to more recent days. Our model also included a dummy variable for the day of the week, distinguishing between regular weekdays (from Monday to Friday excluding holidays), weekends (Saturday and Sunday) and holiday. We found that, using Monday as a reference, there are fewer hospital visits on Tuesday, and the number gradually increases during the week until Saturday. The number of expected visits on a Saturday is comparable to a Monday. Fewer hospital visits are associated with Sunday and bank holidays.

To define the holiday indicator, we accounted for both nationwide holidays and cantonal holidays specific for Bern. For the canton of Bern, the following dates were coded as holidays: New Year’s Day (Jan 1st), Berchtold's Day (Jan 2nd), Good Friday, Easter Monday, Ascension Day, Pentecost Monday, Swiss National Day (Aug 1st), Christmas Day (Dec 25th), St. Stephen Day (Dec 26th).

**Figure S2**. Left panel: estimated coefficients for the autoregressive terms (defined in the Methods as $\beta_{s}$with $s=1,...,7$). Right panel: estimated coefficients for the dummy variables describing the weekly pattern (defined in the Methods as as $\delta_{d}$); Monday is used as a reference.

In Fig. S3, we show the results of the model fitted to the training data. The 1-step ahead model predictions are able to follow the trends in the data, with a short delay.

**Figure S3.** **Model fit on the training set.** For each year in the training set, we show the daily observations (black dots) and model estimates (1-step ahead prediction, green dots).

Weekly rolling mean for both time-series is shown with a continuous line, as a guide for the eye.

In Table S2, we show the root-mean-squared error computed between the 1-step ahead point prediction of the model and the observations. We computed this metric on both the training and the test set. We compared the results of our model with a model neglecting temperature and two last-observation-carried-forward (LOCF) models, with 1-day or 7-day lag. We found that the error of our model was much lower than both LOCF models. The error was similar compared to a model with the same set of predictors but removing temperature. To make a fair comparison of the RMSE across train set (2014-2021) and test set (2022, during the COVID-19 pandemic), we distinguished between the subset of training data which refer to the pre-pandemic period (prior to 2020) and the period of the COVID-19 pandemic (2020-2021).

|  | **RMSE** | | | |
| --- | --- | --- | --- | --- |
| **Model** | **Train set (2014-2021)** | **Train set (2014-2019)** | **Train set**  **(2020-2021, COVID-19)** | **Test set**  **(2022, COVID-19)** |
| Proposed model | 12.37 | 11.21 | 15.35 | 17.36 |
| Proposed model without temperature | 12.67 | 11.48 | 15.71 | 16.87 |
| LOCF (7 day lag) | 17.82 | 16.00 | 22.43 | 18.09 |
| LOCF (1 day lag) | 17.60 | 16.53 | 20.48 | 25.75 |

**Table S2.** Root-mean-squared-error (RMSE) between the 1-step ahead point predictions of different models and the daily observations. The RMSE is stratified by training and test set, and further stratified distinguishing the training set prior or during the COVID-19 pandemic.

For completeness, we report in Table S3 the RMSE on different test sets, obtained by training the model on all data excluding the test set (cross validation across years).

|  | RMSE | | | | | | | | |
| --- | --- | --- | --- | --- | --- | --- | --- | --- | --- |
|  | Test set 2014 | Test set 2015 | Test set 2016 | Test set 2017 | Test set 2018 | Test set 2019 | Test set 2020 | Test set 2021 | Test set 2022 |
| Proposed model | 10.94 | 12.14 | 10.61 | 12.98 | 11.01 | 13.12 | 16.78 | 15.61 | 17.36 |
| Proposed model without temperature | 11.08 | 11.74 | 10.46 | 13.17 | 10.89 | 13.30 | 16.92 | 15.38 | 16.87 |

**Table S3.** Root-mean-squared-error (RMSE) between the 1-step ahead point predictions of different models and the daily observations, under cross validation. For each choice of test set, we train the model on all data except the test year.

### Residual diagnostics

In this section, we report diagnostics plots for the proposed model, including plot with residuals vs. fitted values, QQ plot and autocorrelation function (ACF) on the residuals.

Fig. S4 shows that the conditions on homoscedasticity and normality are met: the residuals are scattered evenly around 0, and they mostly follow a normal distribution, with only slight deviations toward the right tail.

**Fig. S4**. Left: plot with residuals vs. fitted values. Right: residual QQ plot.

We checked for residual autocorrelation for the proposed model. The autocorrelation function on the residuals is illustrated in Fig. S5. The Ljung-Box test with lag = 14 gave a p-value = 0.9. These results show that there is no evidence for residual autocorrelation (p-value = 0.9 with Box-Ljung test up to lag 14), suggesting that the proposed model is well specified.

**Fig S5.** Autocorrelation function of the residuals for the proposed model.

### Exposure-response function with cross-validation

In Fig. 2 in the main text, we showed the exposure-response function estimated using data from 2014 to 2021 as a training set, excluding data for 2022. In this section, we present the estimated exposure-response function resulting from the inclusion of all available years, or exclusion of one single year other than 2022. This cross-validation analysis shows that the shape of the exposure-response function is fairly robust. The major differences are found in the estimate of the right-end tail (mean temperature above 25 °C) when excluding data from 2019 or 2015 from the training set. This is due to the fact that these years correspond to the seasons who registered the highest number of days with high temperature (Fig. S1), hence removing these data from the training sets has an impact on the estimates of the right-end tail of the exposure-response function.

**Figure S6. Estimated non-linear association with temperature, after cross validation on the training set. (a)** Cumulative exposure-response function presented in the main text (Fig. 2a) obtained excluding data for 2022 from the training set. **(b)** Cumulative exposure-response function obtained using all available data (2014-2022) as training set. **(c-j)** Cumulative exposure-response function obtained using all data except one year (either 2021, 2020, …, or 2014).

### Other temperature indicators as exposure variable

In this section, we provide the results of the estimated association between hospital visits and temperature, using different temperature indicators. In the main text, we used mean temperature per day. Notably, we found that replacing the exposure indicator with mean temperature during the day (6am - 6pm) or during the night (6pm - 6am), or maximum temperature, led to similar results in terms of the shape of the estimated cumulative exposure-response function. In contrast, using minimum temperature as the exposure indicator led to highly uncertain results, suggesting that this indicator does not capture relevant heat effects.

**Figure S7**. **Cumulative exposure-response function** obtained using different temperature indicators: **(a)** mean temperature (as in the main analysis); **(b)** mean temperature during the day (6am - 6pm); **(c)** maximum temperature; **(d)** nocturnal mean temperature (6pm - 6am); **(e)** minimum temperature.

### Gaussian and quasi-Poisson regression

We fitted the regression model assuming a Gaussian distribution, which is the most common choice in forecasting models. Studies focusing on estimating the association with temperature (however, without making forecasts), usually adopt a quasi-Poisson distribution[^15^](https://www.zotero.org/google-docs/?8gAx4W). We found that the shape of the exposure-response function estimated from our dataset was not sensitive to the choice of the distribution (Gaussian or quasi-Poisson, Fig. S8); besides, our time-series included large daily counts (more than 100). For these reasons, we decided to use a Gaussian distribution. However, we acknowledge that this may not be a suitable choice when dealing with a time-series with very low case numbers.

**Fig. S8**. **Cumulative exposure-response function** obtained using different distribution families. **(a)** Model assuming a Gaussian distribution (as in the main analysis) **(b)** Model assuming a quasi-Poisson distribution.

### Forecast errors

In Fig. 4, we visually compared the forecast errors for a model with or without temperature, performing cross validation. Here, we report the values median RMSE, along with 2.5% and 97.5% quantiles for the two models (Table S4). Additionally, we report the RMSE obtained using a weekly moving average as a naive k-step ahead forecast model.

**Table S4**. Median RMSE (across all forecast windows) along with 2.5%, 97.5% quantiles for the models shown in Fig. 4.

|  |  | Median RMSE (2.5%, 97.5% quantiles) | |
| --- | --- | --- | --- |
| Forecast horizon | Model | All windows (Fig. 4a) | Windows with average temperature above 22°C (Fig. 4b) |
| 1st week | with temperature | 12.35 (5.95, 24.72) | 11.65 (6.91, 26.34) |
|  | without temperature | 12.41 (5.88, 24.31) | 13.04 (6.37, 25.36) |
|  | naive (weekly moving average) | 13.32 (6.38, 25.55) | 13.50 (6.96, 24.51) |
| 2nd week | with temperature | 12.79 (5.68, 26.55) | 11.31 (6.89, 26.08) |
|  | without temperature | 13.00 (5.97, 25.99) | 12.58 (7.37, 24.10) |
|  | naive (weekly moving average) | 14.11 (6.72, 30.04) | 13.85 (8.64, 24.87) |
| 3rd week | with temperature | 13.53 (6.24, 28.29) | 12.06 (6.54, 26.14) |
|  | without temperature | 14.02 (6.23, 27.39) | 13.47 (8.34, 24.35) |
|  | naive (weekly moving average) | 15.15 (7.20, 43.05) | 15.24 (8.78, 25.93) |

### Observed vs forecasted temperature data

We had access to forecasted temperature data for summer 2022. These forecasts were generated through the COSMO model at MeteoSwiss, providing forecasts up to 6 days ahead. For each forecast reference time (100 days, from 1st of June 2022 to 31st of August 2022), we considered temperature quantiles (10th, 20th, 50th, 80th & 90th) for each lead-time from +0 days to +5 days, for the air temperature at 2m above ground at the Zollikofen station. Post-processing on the gridded ensemble forecast was done as follows: we interpolated the temperature grid to only the Zollikofen measurement station; we computed the quantiles from the 21 ensemble members; we took the daily means (as the original data is hourly). A sample of the data for the first two weeks in June 2022 is illustrated in Fig. S9. The quantile intervals generally correspond to variations of 2 °C compared to the median value.

**Fig. S9. Forecasted temperature data.** Lines indicate median values, shaded areas indicate quantiles (20th - 80th and 10th - 90th). Each color refers to a given reference time, and contains 6 lead times (from +0 days to +5 days).

First, we compared the forecasted temperature data with the observed temperature data used in our main analyses, registered from the Zollikofen station. We found that the median forecasted temperature data are aligned quite well with the observed temperature data (Fig. S10). The best alignment is found for a lead time of 0 days, but good agreement is also found up to a lead time of 4 days. For lead time equal to 5 days, the forecasted temperature data underestimate the observed temperature data.

**Fig. S10. Comparison between (median) forecasted temperature data and observed temperature data.** For each panel, we show a scatter plot with the observed temperature (x-axis) and the corresponding forecasted temperature (y-axis) obtained with a given lead time (from 0 to 5 days in advance).

Secondly, we generated hospital forecasts using as input the median forecasted temperature, compared to using observed temperature. To quantify the impact of the uncertainty in the forecasted temperature, we also generated hospital forecasts using the upper or lower quantiles of the forecasted temperature. As expected, the number of hospital visits are generally slightly higher when using the upper bound of the forecasted temperature instead of median values (Fig. S11). However the upper range of the prediction interval does not increase largely. In other words, the prediction interval for the hospital visits obtained with the median temperature already captures most of the uncertainty, and including uncertainty in the temperature values in input does not lead to a significant extension of the prediction interval.

**Fig. S11. Hospital forecasts using observed vs forecasted temperature data as input. Dots represent observed data of emergency room visits (ERVs).** Black line indicates the 1-step ahead prediction for reference. Colored lines indicate 6-days forecasts obtained in six different time windows, obtained using as predictor the median forecasted temperature (green), the 10% quantile (orange) or the 90% quantile (red). Continuous line indicate the point-prediction, dashed lines indicate the 90% prediction interval.

We computed the RMSE of the point predictions generated by three models (using median, upper or lower quantiles of the forecasted temperature as input), and compared them with the original model using observed temperature data. We found that the average RMSE over 6-days rolling forecast windows remained quite similar across model inputs (Fig S12).

**Fig. S12. Forecast errors using observed or forecasted temperature as input.** Root-mean-squared-error of hospital forecasts against observed hospital data, evaluated in time windows with a length of 6 days, obtained with a model using 10% quantile, 90% quantile or median of forecasted temperature, or observed temperature.
